# 

**Published:** 1898-12-03

**Authors:** 


					\THE HOSPITAL. ? The standard of highest purity."?Lancet. Dko. 3rd, 189$.
CADBURY'S O to* CADBURY'S
OOOOA Jul OOOOA
ABSOLUTELY PURE, NO DRUGS OR ALKALIES USED,
\T_
which uasfUAL
636. Vol. XXY. [rSpTpe"] Saturday,
Dec. 3rd, 1898.
SubnonptaonTorm?,j p<BlCE TWOPBJJCE.

				

